# Dysphagia as the Initial Manifestation of Anti‐HMGCR Antibody–Positive Immune‐Mediated Necrotizing Myopathy Complicated by Pulmonary Embolism

**DOI:** 10.1155/crrh/2235175

**Published:** 2026-11-03

**Authors:** Rohini Kalluri, Elena Ciofoaia, Omobolanle Adetimehin, Aryan Shiari, Igor Dumic, Margherita Milone, Juan Vera Gomez, Erik Sviggum, Celestine Odenigbo, Cristian Madrid, Charles W. Nordstrom

**Affiliations:** ^1^ Department of Family Medicine Mayo Clinic Health System Eau Claire Wisconsin USA mayoclinichealthsystem.org; ^2^ Department of Rheumatology Mayo Clinic Health System Eau Claire Wisconsin USA mayoclinichealthsystem.org; ^3^ Department of Hospital Internal Medicine Mayo Clinic Health System Eau Claire Wisconsin USA mayoclinichealthsystem.org; ^4^ Department of Pulmonary and Critical Care Medicine Mayo Clinic Health System Eau Claire Wisconsin USA mayoclinichealthsystem.org; ^5^ Department of Neurology Mayo Clinic Rochester Minnesota USA mayo.edu; ^6^ Department of Radiology Mayo Clinic Health System Eau Claire Wisconsin USA mayoclinichealthsystem.org

## Abstract

**Background:**

Anti–3‐hydroxy‐3‐methylglutaryl–coenzyme A reductase (anti‐HMGCR) antibody–positive immune‐mediated necrotizing myopathy (IMNM) is a rare autoimmune myopathy characterized by progressive proximal muscle weakness and markedly elevated creatine kinase levels. Although commonly associated with prior statin exposure, statin‐naïve cases and atypical presentations are well described. Patients with idiopathic inflammatory myopathies are also at increased risk for venous thromboembolism (VTE), though data specific to IMNM remain limited.

**Case Presentation:**

An 83‐year‐old man with brief, remote statin exposure presented with progressive proximal muscle weakness and a three‐year history of oropharyngeal dysphagia preceding limb involvement. Laboratory evaluation revealed markedly elevated creatine kinase and transaminases. Anti‐HMGCR antibodies were positive, and muscle biopsy confirmed necrotizing myopathy. During evaluation for occult malignancy, bilateral pulmonary emboli were incidentally identified. The patient required high‐dose corticosteroids, mycophenolate mofetil, and intravenous immunoglobulin for refractory disease. With aggressive immunosuppressive therapy and rehabilitation, he demonstrated substantial clinical recovery at 6 months.

**Conclusion:**

This case highlights oropharyngeal dysphagia as an early and, for a prolonged period, isolated manifestation of anti‐HMGCR antibody–positive IMNM; here, it preceded the onset of limb weakness by approximately 3 years. It underscores the importance of comprehensive diagnostic evaluation in patients with unexplained oropharyngeal dysfunction, in whom an inflammatory myopathy may otherwise be overlooked for years. Clinicians should maintain awareness of thrombotic risk in inflammatory myopathies, particularly during active disease. Early recognition and prompt immunosuppressive therapy remain critical to optimize outcomes and prevent irreversible muscle damage.

## 1. Introduction

Immune‐mediated necrotizing myopathy (IMNM) has three serological subtypes: anti–3‐hydroxy‐3‐methylglutaryl–coenzyme A reductase (HMGCR), anti–signal recognition particle (SRP)–associated antibodies, and seronegative disease [1–4]. Anti‐HMGCR antibody–positive IMNM is a rare and distinct myopathy characterized by progressive muscle weakness and markedly elevated serum creatine kinase (CK) levels. Muscle biopsy typically demonstrates myofiber necrosis with minimal or no inflammatory infiltrate [1–4].

Anti‐HMGCR antibody–positive IMNM is most often associated with prior statin exposure, although statin‐naïve cases are well described [5–8]. The estimated disease prevalence is approximately 3 cases per million people, with an annual incidence of 1–2 cases per million per year [1–8]. The presence of anti‐HMGCR autoantibodies serves as a key diagnostic biomarker, targeting the 3‐hydroxy‐3‐methylglutaryl–coenzyme A (HMG‐CoA) reductase enzyme, which plays a central role in the cholesterol biosynthesis pathway. In genetically or environmentally predisposed individuals exposed to statins, upregulation of HMGCR in regenerating muscle fibers may trigger an autoimmune response that persists despite statin discontinuation, leading to ongoing muscle injury [1–8].

Patients with idiopathic inflammatory myopathies (IIMs) appear to have an increased risk of venous thromboembolism (VTE), but evidence specific to IMNM and anti‐HMGCR antibody–positive disease remains limited. Most available studies evaluate IIM as a broader group, including dermatomyositis, polymyositis, IMNM, and other subtypes [9–11]. Within the IIM spectrum, dermatomyositis carries the most consistently reported association with malignancy and VTE [9–11]. In IMNM, thrombotic risk is biologically plausible because severe active disease can be accompanied by systemic inflammation, profound weakness, immobility, and exposure to immunosuppressive therapy [9–11].

In addition to proximal limb weakness, involvement of the oropharyngeal skeletal muscles represents an important consideration, as it confers an increased risk of dysphagia, aspiration, and dysphonia. Dysphagia typically occurs alongside or after proximal limb weakness in anti‐HMGCR antibody–positive IMNM and only rarely dominates the presentation. In a multinational Australian and UK cohort of 109 anti‐HMGCR cases, dysphagia was documented in 18 of 102 patients (17.6%) [7]. Dysphagia that precedes limb weakness by years is distinctly unusual, and because isolated swallowing difficulty is readily attributed to more common oropharyngeal, esophageal, or neurodegenerative causes, it may lead to prolonged diagnostic delay. This case is noteworthy because it combines two uncommon clinical features of anti‐HMGCR antibody–positive IMNM: oropharyngeal dysphagia that was the sole manifestation of disease for approximately 3 years before any limb weakness appeared and the presence of clinically silent pulmonary embolism at the time of diagnosis. We present this case to highlight the diagnostic challenges associated with atypical presentations of IMNM and to emphasize the importance of considering both dysphagia and VTE as potential manifestations of active disease.

## 2. Case Presentation

An 83‐year‐old White man was admitted following a fall at home precipitated by progressive generalized weakness. He reported a gradual onset of proximal lower extremity muscle weakness over the 3 months preceding admission. His past medical history was notable for well‐controlled Type 2 diabetes mellitus, familial essential tremors, subclinical hypothyroidism, and hyperlipidemia. He was treated 4 years prior to current admission for hyperlipidemia with 20 mg of atorvastatin for about 1 month. He then discontinued the medication on his own because of concern about potential side effects, although he had experienced none at that time. He had no prior history of rheumatologic disease, malignancy, or VTE. He denied alcohol use and illicit drug use but reported chewing tobacco. Family history was negative for hereditary hypercoagulable states, rheumatologic disorders, or inherited malignancies. His home medications included glipizide 7.5 mg daily, primidone for essential tremor, and over‐the‐counter multivitamins.

In addition to generalized muscle weakness, which was more pronounced in the lower extremities, the patient reported a history of oropharyngeal dysphagia that began approximately 3 years prior to the onset of muscle weakness. The dysphagia was intermittent and affected both solids and liquids, with episodes becoming more frequent and the intervals between them shortening over those 3 years. Because the dysphagia was intermittent and was not associated with aspiration or odynophagia, no further evaluation was undertaken during the 3 years preceding the fall and the current admission, in keeping with the patient’s wishes. He denied syncope, palpitations, dyspnea, cough, chest pain, gastrointestinal symptoms, fever, or chills. Notably, he reported an unintentional weight loss of approximately 60 pounds over the preceding year (admission BMI 24.5 kg/m^2^).

On physical examination, strength testing was notable for significant proximal muscle weakness, predominantly affecting the lower extremities, with relative preservation of distal strength. Manual muscle testing demonstrated bilateral shoulder abduction 4/5, elbow extension 4/5, wrist extension 4/5, finger extension 4/5, finger abduction 4/5, hip flexion 2/5, knee extension 4/5, ankle dorsiflexion 5/5, and plantar flexion 5/5. Cranial nerve examination was normal, as was sensory examination. Deep tendon reflexes were intact. Cardiopulmonary and abdominal examinations were unremarkable, and there was no evidence of skin rash or inflammatory arthritis.

Initial laboratory evaluation, including complete blood count and comprehensive metabolic panel, was within normal limits except for elevated aspartate aminotransferase (AST) at 120 U/L and alanine aminotransferase (ALT) at 125 U/L. Gamma‐glutamyl transferase (GGT) levels were within normal limits. CK was markedly elevated at 4972 U/L (normal < 308 U/L). Given the concern for myositis in the setting of significant weight loss, computed tomography (CT) of the chest, abdomen, and pelvis was obtained to evaluate for occult malignancy. Imaging incidentally revealed bilateral pulmonary emboli without right heart strain (Figures 1 and 2), and a continuous heparin infusion was initiated. No findings suggestive of malignancy or focal infection were identified.

**FIGURE 1:**
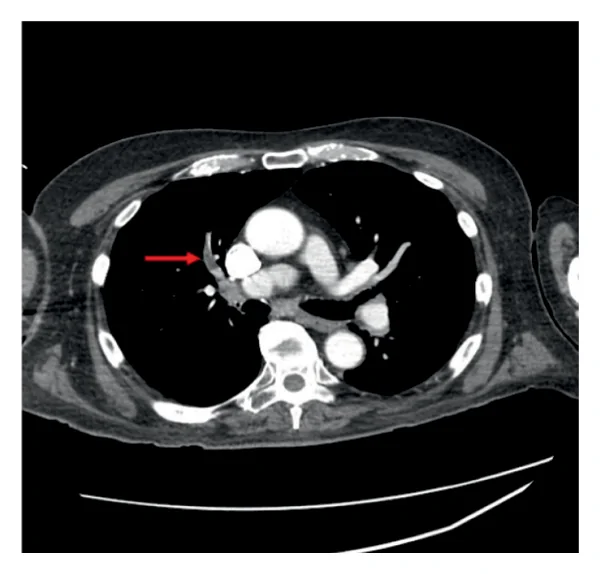
CT pulmonary angiography (axial view) demonstrating acute pulmonary embolism. A contrast‐opacified pulmonary artery in the right upper lobe shows a focal intraluminal filling defect (red arrow), consistent with an acute pulmonary embolus.

**FIGURE 2:**
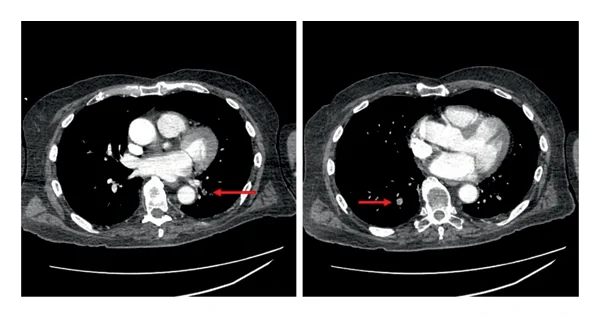
CT pulmonary angiography demonstrating bilateral lower‐lobe pulmonary emboli. Axial images show central intraluminal filling defects within segmental and subsegmental pulmonary arteries of the right and left lower lobes (arrows), with surrounding contrast opacification, consistent with acute pulmonary embolism.

Due to dysphagia, the patient underwent a speech‐language pathology evaluation, which demonstrated oropharyngeal dysphagia with prolonged mastication, reduced hyolaryngeal excursion, and wet vocal quality following oral intake. Esophagogastroduodenoscopy revealed no evidence of esophageal lesions or infection. Despite intravenous fluid administration, CK levels failed to improve. In the context of persistently elevated CK and transaminases, as well as muscle weakness, an inflammatory myopathy was suspected.

Further evaluation included magnetic resonance imaging (MRI) of the thighs, aldolase level, myositis‐specific antibody panel, myositis‐associated antibody panel, and cytosolic 5′‐nucleotidase 1A (cN1A) antibody testing. Anti–HMG‐CoA reductase antibody levels were elevated, supporting a diagnosis of IMNM. The other tested antibodies were negative. MRI of the thighs demonstrated nonspecific muscle signal abnormalities involving the right greater than left hip adductors and hamstrings, as well as bilateral rectus femoris muscles with heterogeneous midmuscle enhancement (Figures 3 and 4).

**FIGURE 3:**
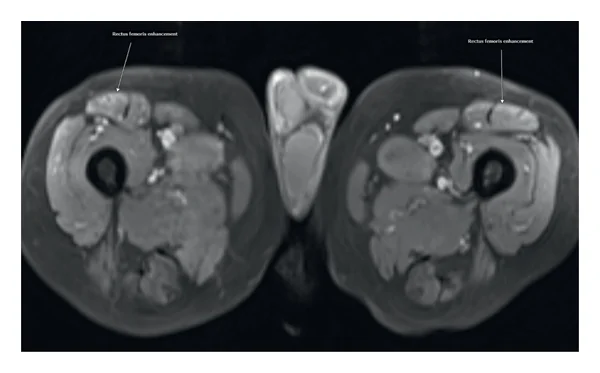
Coronal T2‐weighted fat‐suppressed MRI of the thighs demonstrating increased signal intensity involving the hip adductors and hamstrings, consistent with inflammatory myopathy (arrows).

**FIGURE 4:**
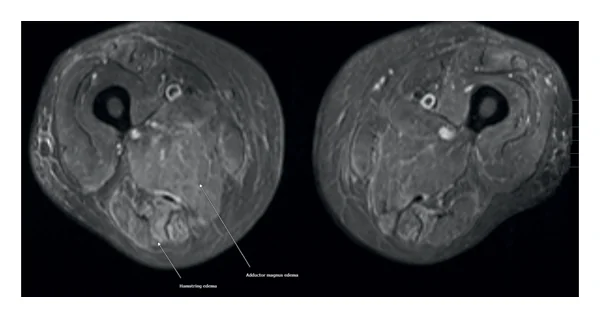
Axial postcontrast MRI demonstrating heterogeneous enhancement of the bilateral rectus femoris muscles, consistent with active myositis (arrows).

Serologic testing was negative for antinuclear antibody, cyclic citrullinated peptide antibody, rheumatoid factor, antineutrophil cytoplasmic antibodies, myeloperoxidase, proteinase 3, and complement component 3. The patient underwent muscle biopsy of the rectus femoris without complication. He was initiated on prednisone 60 mg daily for a provisional 2‐week course pending outpatient rheumatology follow‐up. *Pneumocystis jirovecii* pneumonia prophylaxis with trimethoprim–sulfamethoxazole and gastrointestinal prophylaxis with pantoprazole were also initiated. Apixaban was initiated for ongoing treatment of PE and was continued for 6 months after discharge.

The patient was evaluated in clinic approximately 3 weeks after discharge. Muscle biopsy demonstrated an active and chronic myopathy with numerous necrotic fibers, regenerating fibers, mild increase in endomysial connective tissue, and mild focal perivascular inflammatory reaction (Figure 5). At his initial outpatient rheumatology follow‐up, the patient reported minimal clinical improvement in muscle strength and dysphagia. He was unable to ambulate independently and required a wheelchair. He also reported ongoing choking episodes with both solids and liquids, accompanied by a worsening cough. Mycophenolate mofetil was initiated as a steroid‐sparing agent; however, because of progressive symptoms and severe dysphagia with aspiration risk, he also received intravenous methylprednisolone 500 mg twice daily for 3 days and intravenous immunoglobulin (IVIG) at a total dose of 2 g/kg based on ideal body weight over 5 days while mycophenolate mofetil was continued. Following transition back to oral corticosteroids, CK levels began to rise again. Therapy was therefore escalated to mycophenolate mofetil 1.5 g twice daily, a gradual prednisone taper, continued monthly 5‐day IVIG cycles, and intensive physical therapy with submaximal exercise.

**FIGURE 5:**
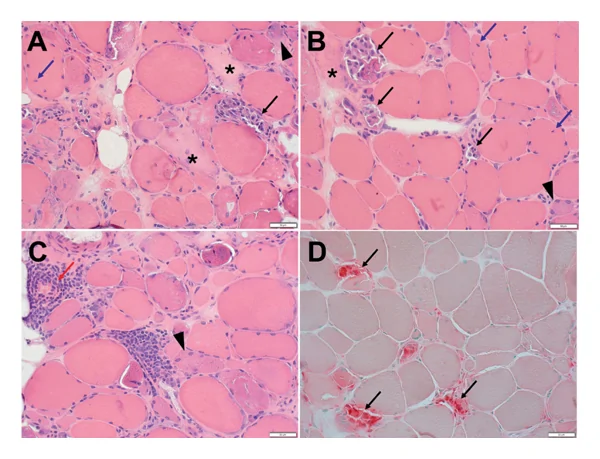
Biopsy of the rectus femoris. (A–C) Hematoxylin–eosin stained sections showing muscle fiber size variability, a mild increase in nuclei internalization (blue arrows), several necrotic fibers (asterisks), some of which are invaded by macrophages (black arrows), regenerating fibers (arrowheads), and a patchy mild increase in endomysial connective tissue in between muscle fibers suggesting a component of chronicity. A small collection of perivascular inflammatory cells is present at a perimysial site (C, red arrow). (D) Acid phosphatase–stained section showing red macrophages (arrows) invading some necrotic fibers.

At 6‐month follow‐up, the patient demonstrated substantial symptomatic improvement. He returned to his baseline functional status, ambulated independently without a walker, and experienced complete resolution of the dysphagia. Laboratory evaluation showed normalization of his CK, aldolase, AST, and ALT levels.

## 3. Discussion

This case report describes an elderly White man who developed anti‐HMGCR antibody–positive IMNM approximately 4 years after a brief, remote exposure to atorvastatin. Its defining feature is that oropharyngeal dysphagia was the presenting symptom and remained the sole manifestation of disease for approximately 3 years before proximal limb weakness became apparent, an interval considerably longer than that described in previously reported dysphagia‐predominant cases [12–16]. By contrast, patients with anti‐HMGCR antibodies typically present with subacute or chronic progressive proximal muscle weakness, often accompanied by myalgia or fatigue, and the diagnosis rests on a combination of clinical evaluation, serologic testing for anti‐HMGCR antibodies, electromyography (EMG), and muscle biopsy. In this patient, quantitative anti‐HMGCR antibody titers were not available, and only qualitative positivity could be reported; this is a limitation because titers correlate broadly with disease activity and treatment response.

Dysphagia is a recognized feature of anti‐HMGCR antibody–positive IMNM but is rarely its initial manifestation, since progressive symmetric proximal muscle weakness usually predominates at presentation [8]. Reported frequencies vary considerably with case ascertainment: Dysphagia was documented in 18 of 102 patients (17.6%) in a multinational Australian and UK cohort [7] and in 4 of 13 anti‐HMGCR antibody–positive patients (31%) in a largely statin‐naïve Swedish cohort [6]. In one Chinese cohort, it was observed in approximately half of anti‐HMGCR antibody–positive patients, although most of those patients developed dysphagia against a background of established muscle weakness rather than as the primary symptom [17]. In the present patient, the sequence was reversed: Intermittent dysphagia to both solids and liquids, increasing in frequency over 3 years, was the only manifestation of disease, and the diagnosis was established only after a fall brought progressive proximal weakness to attention.

It is biologically plausible that the swallowing musculature can be affected selectively and well in advance of the limbs. In an autopsy‐proven case of anti‐HMGCR IMNM, necrotizing myopathy of the pharyngeal muscles was severe, while thigh muscle involvement was only mild, establishing that the pharyngeal muscles may be affected disproportionately [13]. A diagnostic algorithm study of 72 patients referred with unexplained dysphagia as the leading or only symptom reported a mean symptom duration of 3.8 ± 2.9 years before evaluation, and 19 of these patients (26%) were ultimately diagnosed with an IIM; most had subclinical involvement of muscles outside the oropharynx, although in some no extra‐oropharyngeal involvement could be demonstrated [12]. That cohort included patients with inclusion body myositis, dermatomyositis, and polymyositis rather than anti‐HMGCR IMNM, but the multiyear latency it documents closely parallels the 3‐year interval observed here and illustrates how readily isolated dysphagia is ascribed to more common causes. The overall prevalence of dysphagia in IIMs is estimated at approximately 36% and reflects involvement of the swallowing musculature, including reduced pharyngeal contractility, cricopharyngeal dysfunction, and impaired laryngeal elevation [12]. The course in the present patient is consistent with a slowly progressive, initially subclinical myopathy in which the pharyngeal muscles crossed the threshold of clinical dysfunction years before the limb muscles did.

Previously reported dysphagia‐predominant anti‐HMGCR IMNM has been characterized by short latency and high morbidity. In an autopsy‐proven case, dysphagia progressed to fatal aspiration pneumonia within 3 weeks of symptom onset, and the diagnosis was established only postmortem [13]. In another, IMNM was diagnosed approximately 7 months after the onset of dysphagia, during evaluation for aspiration pneumonia [14]. A case series described two patients in whom dysphagia was a prominent clinical feature; both also exhibited the unusual finding of subacute ophthalmoparesis [15]. Dysphagia of 3 weeks’ duration was likewise the presenting symptom in a patient with concurrent IMNM and autoimmune hepatitis, who required prolonged enteral feeding [16]. Set against these reports, the 3‐year prodrome in the present case appears substantially longer than any previously described interval between dysphagia onset and limb weakness in anti‐HMGCR antibody–positive IMNM, and the complete recovery of swallowing function contrasts with the fatal and protracted courses reported elsewhere.

The incidental finding of bilateral pulmonary emboli also warrants comment. Patients with IIMs carry a four‐ to eightfold higher risk of VTE than the general population [9, 10], with the risk concentrated near disease onset and greatest during the first year after diagnosis [11, 18–20]; among subtypes, dermatomyositis is the most consistently implicated [20]. VTE is not, however, an established extramuscular manifestation of anti‐HMGCR IMNM [21]. In this patient, the emboli were detected during the early active phase of disease, when systemic inflammation, severe proximal weakness, and markedly limited mobility were all present; they are therefore best interpreted as a clinically important complication arising in that setting rather than as evidence of a disease‐specific thrombotic phenotype. Antiphospholipid antibody testing, including lupus anticoagulant, anticardiolipin antibodies, and anti‐beta‐2 glycoprotein I antibodies, was not performed. This is a limitation because antiphospholipid syndrome remains an important differential consideration in otherwise unexplained VTE.

Treatment of anti‐HMGCR antibody–positive IMNM typically includes IVIG, high‐dose corticosteroids, and additional immunosuppressive agents such as methotrexate, azathioprine, or mycophenolate mofetil. Responses to therapy are variable, and relapse is common during tapering of immunosuppression [1–8, 22]. Life‐threatening manifestations, including profound weakness or dysphagia with aspiration risk, are associated with increased risk of morbidity and mortality.

Initial triple immunosuppressive therapy in this setting has demonstrated high efficacy, with approximately 10% of patients failing this induction strategy. Delayed initiation of treatment independently predicts worse outcomes, with treatment delay associated with lower odds of successful maintenance immunosuppression and prolonged time to first remission [23]. Nearly 90% of patients who successfully discontinued glucocorticoids were treated with triple immunosuppressive regimens. Some patients recover full strength despite persistently elevated CK levels, suggesting attenuated disease in which muscle regeneration outpaces destruction. Conversely, muscle weakness may persist after CK normalization in patients whose disease has been undertreated over the long term and in whom permanent fatty replacement of muscle has developed [8, 22].

## 4. Conclusion

This case highlights the importance of comprehensive diagnostic evaluation in patients presenting with unexplained oropharyngeal dysphagia, which may be the earliest—and, for a prolonged period, the only—manifestation of an underlying autoimmune myopathy. The 3‐year interval between the onset of dysphagia and the appearance of proximal muscle weakness in this patient is, to our knowledge, among the longest reported in anti‐HMGCR antibody–positive IMNM, and it illustrates how readily such a presentation escapes recognition. Clinicians should also recognize that patients with IIM have an increased risk of VTE, particularly during early active disease, while data specific to anti‐HMGCR antibody–positive IMNM remain limited.

## Funding

This research received no external funding.

## Disclosure

This research was conducted as part of the authors’ institutional clinical practice.

## Ethics Statement

This case report was conducted in accordance with the ethical standards of the institutional research committee and with the principles of the Declaration of Helsinki. Institutional review board approval was not required for this single‐patient case report according to institutional policy.

## Consent

Written informed consent for publication of this case report and any accompanying images was obtained from the patient. A copy of the written consent form is available for review by the journal upon request.

## Conflicts of Interest

The authors declare no conflicts of interest.

## Data Availability

Data sharing is not applicable to this article because no new datasets were generated or analyzed. All relevant clinical information supporting the findings of this case report is included within the published article.
